# Evaluating the Role of α‐Synuclein Seed Amplification as a Disease Progression Marker: Evidence and Uncertainties

**DOI:** 10.1002/mdc3.70434

**Published:** 2025-11-14

**Authors:** Daniel Belete, Christian Mattjie, Brook Huxford, Jonathan P. Bestwick, Alastair J. Noyce, Cristina Simonet

**Affiliations:** ^1^ Centre for Preventive Neurology, Wolfson Institute of Population Health, Faculty of Medicine and Dentistry, Queen Mary University of London London United Kingdom; ^2^ Machine Learning Theory and Application Research Laboratory, Pontifícia Universidade Católica do Rio Grande do Sul Porto Alegre Brazil; ^3^ Department of Neurology Royal London Hospital, Barts Health NHS Trust London United Kingdom; ^4^ Department of Neurology Homerton University Hospital, Homerton University Hospital NHS Foundation Trust London United Kingdom

**Keywords:** Parkinson's disease, α‐Synuclein seed amplification assay, disease progression, RBD

## Abstract

**Background:**

α‐synuclein seeding amplification assay (α‐synuclein SAA) development as a diagnostic biomarker for Parkinson's disease (PD) has shown promising results over the past decade. However, the utility of these assays in the prediction of disease progression is unclear.

**Objectives:**

To assess the relationship between α‐synuclein SAA and PD‐specific clinical outcome measures.

**Methods:**

We extracted longitudinal data on individuals with sporadic PD from the Parkinson's Progression Markers Initiative at baseline and 5 years follow‐up. Primary outcome measures included MDS‐UPDRS Part III, Montreal Cognitive Assessment (MoCA) and L‐dopa equivalent daily dose (LEDD). Secondary outcome measures included REM Sleep Behavior Disorder Screening Questionnaire (RBDSQ) question‐6 and other non‐motor assessments. α‐synuclein SAA kinetic parameters were added to linear regression models to assess their impact on model fit.

**Results:**

We included 279 participants in the final analysis. There was no consistent evidence that α‐synuclein SAA parameters at baseline improved our prediction models for MDS‐UPDRS Part III, MoCA or LEDD at 5 years. α‐synuclein SAA kinetic parameters improved model fit for RBDSQ question‐6 and indicated that fast seeding profiles were associated with higher scores.

**Conclusions:**

We did not find evidence of a relationship between α‐synuclein SAA and disease progression however α‐synuclein SAA was associated with RBDSQ. Further work is needed to understand the factors influencing α‐synuclein aggregation kinetics and the role of α‐synuclein SAA in disease prognosis.

The development of a diagnostic biomarker for synuclein‐related disorders represents a significant milestone in the field of Parkinson's disease (PD) research.^1^, ^2^ α‐synuclein seeding amplification assays (α‐synuclein SAA) have demonstrated a high level of sensitivity and specificity in detecting PD and other Lewy body‐related pathology.^3^, ^4^, ^5^, ^6^ These assays may also distinguish between different synucleinopathies, such as PD and multiple system atrophy.^7^

The utility of α‐synuclein SAA in detecting PD pathology at an earlier stage is currently being investigated. This will have important implications for patients and clinical researchers as it is likely that future trials for disease modifying treatments will incorporate α‐synuclein SAA into their eligibility criteria. Staging frameworks for PD and neuronal α‐synuclein disease have also recently been proposed.^8^, ^9^ α‐synuclein‐based biomarkers play a key role in these frameworks, which aim to accelerate the path to therapeutics through a biological definition of PD. Neurofilament light chain (NfL), another biomarker of interest, is raised in individuals with PD compared to healthy controls.^10^ Although it is a marker of axonal injury that is not specific to PD, some studies have shown it to be an indicator of motor and non‐motor disease progression.^10^, ^11^ Serum NfL is highly correlated with cerebrospinal fluid NfL and as such is a promising candidate as a minimally invasive biomarker.

Given that α‐synuclein SAA is likely to have such an important role in future research, and potentially in clinical practice, it is important to understand its associations with PD progression. In this study we used data from the Parkinson's Progression Markers Initiative (PPMI) to assess the relationship between α‐synuclein SAA parameters and PD‐specific clinical outcome measures. We also conducted a sub‐group analysis to explore the prognostic role of serum NfL.

## Methods

### Dataset

We utilized longitudinal data from the PPMI study. PPMI is an observational, multicenter study designed to explore the progression of clinical features, imaging and biological biomarkers in PD, prodromal PD and healthy controls. PD was confirmed by site investigators and by a consensus committee review. Participants with sporadic PD were within 2 years of diagnosis and were not taking dopaminergic medications for PD at the time of enrolment. Full details of recruitment into PPMI and the study design have been reported elsewhere.^12^ We included participants with sporadic PD who had been recruited between July 2010 and March 2019. Data were extracted in May 2024.

### Outcomes

Participants completed a series of baseline assessments, which were subsequently repeated annually over a 5‐year period. For primary outcome measures, we included data from the Movement Disorder Society Unified Parkinson's Disease Rating Scale (MDS‐UPDRS) Part III, Montreal Cognitive Assessment (MoCA) and L‐dopa equivalent daily dose (LEDD) at the Year 5 study visit. We used off‐state MDS‐UPDRS Part III examinations for participants who were receiving dopaminergic medications. For secondary outcome measures, we assessed non‐motor markers using Scales for Outcomes in Parkinson's disease—Autonomic Dysfunction (SCOPA‐AUT), Benton Judgment of Line Orientation (JLO), Hopkins Verbal Learning Test—Revised (HVLT‐R), Geriatric Depression Scale Short Version (GDS), question‐6 of the REM Sleep Behavior Disorder Screening Questionnaire (RBDSQ) and Epworth Sleepiness Scale (ESS) at the Year 5 study visit. HVLT‐R recall score was calculated by combining the three learning trials, the delayed recall score was taken from the corresponding trial and the Recognition Discrimination Index (RDI) was calculated as the difference between number of correct responses and false positives.

### Biomarkers

Each participant had a cerebrospinal fluid (CSF) sample taken at their baseline assessment. CSF samples were analyzed using two different versions of the Amprion α‐synuclein SAA and qualitative and quantitative α‐synuclein SAA parameters were reported, as previously described.^13^, ^14^ Briefly, the Amprion‐24 h αS‐SAA consisted of 100 mM PIPES pH 6.5, 0.5 M NaCl, 0.1% sarkosyl, 10 μM ThT, 0.3 mg/mL recombinant αSyn, 40 μL CSF and two 1/8″ silicon nitride beads. Samples were run in triplicates in a 96 well plate incubated at 42°C and shaken every 14 minutes. Florescence was measured after each shaking cycle over 24 hours. The Amprion‐αS‐SAA (referred to as the Amprion‐150 h αS‐SAA in this paper) consisted of 100 mM PIPES pH 6.5, 0.5 M NaCl, 10 μM ThT, 0.3 mg/mL recombinant αSyn, 20% CSF v/v and 1 3/32″ silicon nitride bead. Samples were run in triplicates in a 96 well plate incubated at 37°C and shaken every 29 minutes. Florescence was measured after each shaking cycle over 150 hours. We explored the relationship between α‐synuclein SAA kinetic measures and clinical outcome measures. As two separate versions of α‐synuclein SAA have been used in the PPMI dataset, we analyzed the kinetic parameters of the different assays separately. Participants who had either both versions or only one version of the α‐synuclein SAA were included in the analysis. The outcome for each α‐synuclein SAA was reported as positive, negative or inconclusive. An inconclusive outcome was given to borderline samples and where there was a sufficient remaining sample it was reanalyzed. When inconclusive results were reported, we took the result from a definitive repeat. Only participants with assays that demonstrated positive seeding were included. Kinetic parameters included maximum florescence (Fmax), time to threshold (TTT) and area under the curve (AUC) for both assays. Time to reach 50% Fmax (T50) and slope (SLOPE) from the Amprion‐150 h αS‐SAA, and maximum slope (Smax) and time to Smax (TSmax) from the Amprion‐24 h αS‐SAA were also analyzed. As assays were run in triplicate, median for each parameter was used in the statistical analysis. Kinetic parameters were also compared in participants who had both assays conducted on their baseline CSF samples between July 2010 and May 2024.

Serum NfL was selected instead of CSF NfL due to its availability in a greater proportion of the cohort. It also demonstrates a strong correlation with CSF NfL.^15^, ^16^ Serum NfL was measured longitudinally using the Simoa singleplex NF‐light assay in the PPMI cohort. We extracted all available measurements from the baseline study visit. A sub‐group analysis was conducted using baseline serum NfL and PD clinical outcome measures.

### Statistical Analysis

All participants with a baseline α‐synuclein SAA measurement and 5‐year follow‐up were included in the analysis. Welch t‐test was used to compare parametric data. Wilcoxon rank sum test was used to compare skewed baseline data which included disease duration, MDS‐UPDRS Part III, MoCA and LEDD. Categorical variables were compared using Fisher's exact test. A basic linear regression model was built for each primary and secondary outcome measure. This was in the form: Year 5 outcome ~ outcome at baseline + age + sex + disease duration except for MoCA which was in the form: Year 5 outcome ~ outcome at baseline + age + sex + disease duration + years in education. A biomarker model was then built (basic model + biomarker). NfL values were log transformed due to its skewed distribution. The model fit was evaluated using the adjusted R^2^ and Akaike information criterion (AIC). The models were compared using likelihood ratio tests. Participants with missing baseline or follow up outcome data were excluded from the relevant statistical analysis. The Benjamini‐Hochberg procedure was used to correct for multiple comparisons. All statistical analysis was conducted using R V.4.3.3.

## Results

### Demographics and Characteristics

**Figure 1:**
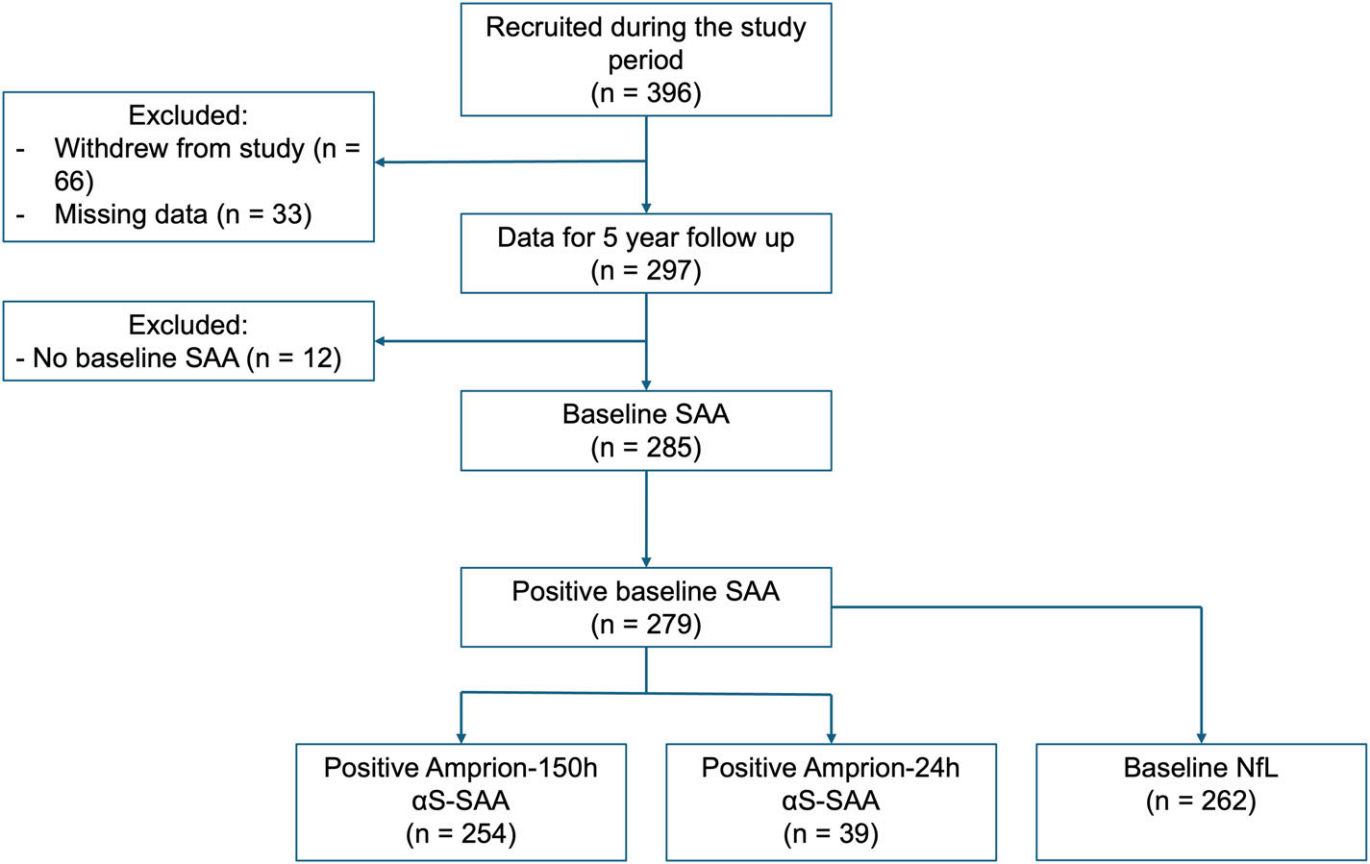
Flow chart showing selection of individuals. This includes individuals who had both versions of the αS‐SAA. α‐synuclein seeding aggregation assay (αS‐SAA), neurofilament light chain (NfL).

A total of 396 participants with sporadic PD were recruited between July 2010 and March 2019 (Fig. 1). 66 participants withdrew from the study prior to their 5‐year follow‐up visit (Table S1) and 33 did not have data recorded for 5‐year follow‐up visit. These participants were excluded from this analysis. Of the 297 participants followed up at 5 years, 285 (96.0%) had a baseline α‐synuclein SAA conducted. Two hundred and sixty‐six participants had baseline CSF conducting using the Amprion‐150 h αS‐SAA and 43 on the Amprion‐24 h αS‐SAA. Most assays (94.8%) had a positive α‐synuclein seeding profile. Four Amprion‐150 h αS‐SAAs had an inconclusive result without a definitive repeat and were excluded. Two hundred and fifty‐four participants on the Amprion‐150 h αS‐SAA and 39 on the Amprion‐24 h αS‐SAA demonstrated α‐synuclein seeding. All participants with the Amprion‐24 h αS‐SAA had Type 1 (Lewy body disease) seeding profiles.

Of the 279 participants with a positive seeding profile, most were male (66.7%) and white (92.8%) and their median age was 62 years (IQR 54–68) (Table 1). Seventy‐seven participants had missing data for off‐state MDS‐UPDRS Part III examinations at year‐5 follow‐up. All these participants, except one, had on‐state examinations indicating they may have been unable to withhold medication long enough to complete the off‐state examination. Nine participants had missing outcome data elsewhere (including participants with multiple areas of missing data) which comprised of two missing results for MoCA, two for SCOPA‐AUT, five for JLO, three for HVLT, one for GDS, one for RBDSQ and two for ESS. Two hundred and sixty‐two of these participants had serum NfL measurements available at baseline and were included in the NfL sub‐group analysis. Median MDS‐UPDRS Part III at baseline was 19 (IQR 14–25) and for MoCA was 28 (IQR 26–29). The median change after 5 years was an increase of 10.5 points (IQR 4–20.75) in the MDS‐UPDRS Part III, no change (IQR −2‐1) in MoCA and an increase of 440 mg (IQR 0 mg‐718.7 mg) in LEDD.

**TABLE 1:** Demographic and clinical information at baseline

Characteristics	*n* = 279
Age (median (IQR))	62 (54–68)
Sex (%)	
Female	93 (33.3)
Male	186 (66.7)
Ethnicity (%)	
White	259 (92.8)
Hispanic or Latino	2 (0.7)
Asian	4 (1.4)
Black/African American	4 (1.4)
American Indian/Alaska Native	1 (0.4)
Multi‐ethnic	9 (3.2)
Education (years) (median (IQR))	16 (14–18)
Disease duration years (median (IQR))	0.33 (0.17–0.67)
Baseline clinical measures	
MDS‐UPDRS Part III	19 (14–25)
MoCA	28 (26–29)
LEDD (mg)	0 (0–0)

Abbreviations: LEDD, levodopa equivalent daily dose; MDS‐UPDRS, Movement Disorder Society‐Unified Parkinson's Disease Rating Scale, Part III (motor section); MoCA, Montreal Cognitive Assessment.

### Basic and Biomarker Linear Regression Models

We assessed if the α‐synuclein SAA kinetic measures improved model fit. Amprion‐150 h αS‐SAA version was the most used assay. Two participants had T50 values provided for only two replicates and were excluded from the relevant model. Adding kinetic parameters to the basic model did not improve model fit for any of our primary outcomes for either assay version (Table 2 and S2). Analysis of kinetic parameters in the secondary outcomes found model fit for RBDSQ question‐6 was improved when TTT (*P* = 1.63e−04), T50 (*P* = 4.59e−04) and AUC (*P* = 1.81e−03) from the Amprion‐150 h αS‐SAA version were added to the basic model(Table S3). In the biomarker models a shorter TTT and T50, and greater AUC were associated with an increased RBDSQ question‐6 score. The association remained following correction for multiple comparisons for TTT (FDR = 0.02) and T50 (FDR = 0.03), however not for AUC (FDR = 0.07). Fmax in the Amprion‐150 h αS‐SAA improved model fit for HVLT‐R delayed recall (*P* = 0.02) and RDI (0.04), and GDS (0.03). None of these associations met the FDR threshold for significance. TTT (*p* = 0.07) and Tsmax (*P* = 0.09) from the Amprion‐24 h αS‐SAA version showed a trend to improve model fit for RBDSQ question‐6.

**TABLE 2:** Summary of basic and biomarker models for each primary outcome measure

Outcome	Basic model		Biomarker model					Likelihood ratio	FDR
	Adjusted R^2^	AIC	Biomarker regression coefficient	Biomarker regression coefficient 95% LCI	Biomarker regression coefficient 95% UCI	Adjusted R^2^	AIC		
Amprion − 150 h αS‐SAA									
UPDRS‐III ~ AUC	2.89E−01	1.39E+03	−4.58E−08	−4.64E−07	3.72E−07	2.85E−01	1.39E+03	8.26E−01	9.91E−01
UPDRS‐III ~ Fmax	2.89E−01	1.39E+03	−1.00E−05	−6.62E−05	4.61E−05	2.86E−01	1.39E+03	7.21E−01	9.19E−01
UPDRS‐III ~ SLOPE	2.89E−01	1.39E+03	6.80E−02	−6.51E−02	2.01E−01	2.89E−01	1.39E+03	3.06E−01	8.62E−01
UPDRS‐III ~ T50	2.89E−01	1.38E+03	5.36E−02	−9.20E−02	1.99E−01	2.87E−01	1.39E+03	4.60E−01	8.62E−01
UPDRS‐III ~ TTT	2.89E−01	1.39E+03	7.54E−02	−6.73E−02	2.18E−01	2.90E−01	1.39E+03	2.90E−01	8.62E−01
MoCA ~ AUC	2.26E−01	1.31E+03	−4.64E−08	−1.47E−07	5.45E−08	2.25E−01	1.31E+03	3.59E−01	8.62E−01
MoCA ~ Fmax	2.26E−01	1.31E+03	−8.63E−06	−2.23E−05	5.03E−06	2.28E−01	1.31E+03	2.08E−01	8.62E−01
MoCA ~ SLOPE	2.26E−01	1.31E+03	−1.32E−02	−4.68E−02	2.04E−02	2.25E−01	1.31E+03	4.32E−01	8.62E−01
MoCA ~ T50	2.26E−01	1.30E+03	1.32E−02	−2.18E−02	4.81E−02	2.25E−01	1.30E+03	4.52E−01	8.62E−01
MoCA ~ TTT	2.26E−01	1.31E+03	9.31E−03	−2.53E−02	4.40E−02	2.24E−01	1.31E+03	5.91E−01	8.82E−01
LEDD ~ AUC	1.88E−02	3.80E+03	7.06E−06	−6.48E−06	2.06E−05	1.90E−02	3.80E+03	3.00E−01	8.62E−01
LEDD ~ Fmax	1.88E−02	3.80E+03	−8.88E−04	−2.72E−03	9.45E−04	1.85E−02	3.80E+03	3.36E−01	8.62E−01
LEDD ~ SLOPE	1.88E−02	3.80E+03	−2.10E+00	−6.60E+00	2.39E+00	1.82E−02	3.80E+03	3.52E−01	8.62E−01
LEDD ~ T50	1.95E−02	3.77E+03	−2.91E+00	−7.57E+00	1.75E+00	2.15E−02	3.77E+03	2.15E−01	8.62E−01
LEDD ~ TTT	1.88E−02	3.80E+03	−1.45E+00	−6.09E+00	3.20E+00	1.63E−02	3.80E+03	5.36E−01	8.82E−01
NfL									
UPDRS‐III	2.59E−01	1.44E+03	−2.78E+00	−7.30E+00	1.74E+00	2.61E−01	1.44E+03	2.18E−01	8.62E−01
MoCA	2.43E−01	1.34E+03	−3.64E−01	−1.48E+00	7.53E−01	2.41E−01	1.34E+03	5.16E−01	8.82E−01
LEDD	1.90E−02	3.91E+03	−5.47E+00	−1.53E+02	1.42E+02	1.52E−02	3.91E+03	9.41E−01	9.91E−01

Abbreviations: AIC, Akaike information criterion; α‐synuclein SAA, α‐synuclein seeding aggregation assay; MDS‐UPDRS, Movement Disorder Society‐Unified Parkinson's Disease Rating Scale, Part III (motor section); MoCA, Montreal Cognitive Assessment; LEDD, levodopa equivalent daily dose; Fmax, maximum florescence; TTT, time to threshold; AUC, area under the curve; T50), time to reach 50% Fmax; SLOPE, slope; Smax, maximum slope; TSmax, time to Smax; NfL, neurofilament light chain.

In the baseline serum NfL sub‐group the biomarker models were not significantly different from the basic models for the primary outcome measures (Table 2). The biomarker model for HVLT‐R delayed recall had an improved fit compared to the basic model (*P* = 0.01). This association did not remain after correction for multiple comparisons (FDR = 0.35). In this model logNfL was negatively correlated with delayed recall scores (Table S3).

### Kinetic Parameters between SAA Versions

**Figure 2:**
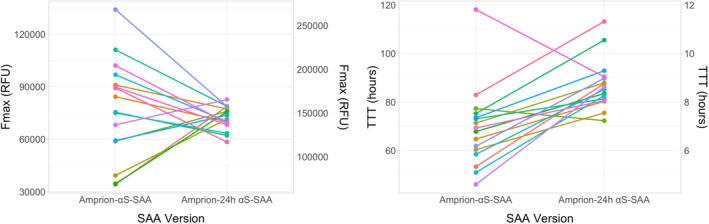
Change in kinetic parameters between the Amprion‐24 h αS‐SAA and Amprion‐150 h αS‐SAA version in the same individual's baseline CSF.

We analyzed kinetic parameters between SAA versions conducted on the same baseline CSF samples. In total we found 16 individuals with sporadic PD recruited up to May 2024 who demonstrated positive seeding and had both assay versions completed on their baseline CSF (Fig. 2). In our linear regression models (Amprion‐150 h αS‐SAA kinetic parameter ~ Amprion‐24 h αS‐SAA kinetic parameter) we did not find evidence that any of the shared kinetic parameters (Fmax and TTT) were associated.

## Discussion

In this study we analyzed the relationship between α‐synuclein SAA and longitudinal changes in MDS‐UPDRS Part III, MoCA and LEDD data in participants with PD. Kinetic parameters from α‐synuclein SAA results did not improve prediction models for these primary clinical outcomes at 5 years. Of interest, analysis of our secondary outcome measures found models for RBDSQ question‐6 score were improved with the addition of TTT and T50. In the biomarker models for RBDSQ, faster kinetic profiles (in this case shorter TTT and T50) were associated with an increased RBDSQ score over 5 years. Serum NfL did not improve models for our primary outcomes however there was improvement with its addition to the HVLT‐R delayed recall model although this did not meet the FDR threshold. Finally, comparing kinetic parameters between the two types of α‐synuclein SAAs showed that kinetic parameters were not consistent between the two versions of the assay.

While the evidence for α‐synuclein SAA as a diagnostic tool for individuals with Lewy body disease is robust, the evidence for α‐synuclein as a disease progression maker is rather more inconclusive. Several studies looking at both α‐synuclein CSF concentration and α‐synuclein SAA kinetics have been published in the past decade. While some studies have found α‐synuclein CSF concentration to be predictive for cognitive decline, others have not been able to replicate these results.^17^, ^18^, ^19^ Similarly, cross‐sectional studies looking at α‐synuclein SAA kinetics and clinical severity markers have found heterogeneous results.^20^, ^21^, ^22^, ^23^ Brockmann and colleagues conducted longitudinal analysis on kinetic parameters and cognitive decline.^24^ Their study found “fast” kinetic parameters (ie, shorter TTT, higher Fmax and AUC) were associated with development of cognitive impairment. There are several reasons we may not have been able to replicate these positive findings. There was a notable difference in average disease duration between the present study and that by Brockmann et al (6 years vs 10–11 years) which could indicate that with a longer follow‐up of the PPMI cohort, differences between participants with “slow” and “fast” kinetic profiles emerge. As demonstrated by Bräuer and collaborators, kinetic properties are influenced by assay protocols, potentially limiting the applicability of kinetic α‐synuclein SAA parameters in diagnosis and prognosis of PD.^23^ They carried out a cross‐sectional analysis and found that different α‐synuclein monomer preparation changed kinetic profiles and their correlation with cognitive assessments. Reagents and buffer solution properties can also have large impacts on seeding kinetics.^25^, ^26^, ^27^ In our analysis we did not find that kinetic parameters of the same baseline CSF sample were associated between assay versions. In addition to assay protocol driven changes, in vitro studies have found components of the CSF milieu to be implicated with aggregation kinetics.^28^ It is therefore possible that the current heterogeneity in α‐synuclein SAAs and CSF collection methods could explain some of the inconsistency in the current literature.

We chose to analyze RBDSQ question‐6 as scores of ≥1 in this subsection have a high sensitivity and specificity for polysomnography confirmed RBD in de novo PD.^29^ The addition of TTT, T50 and AUC improved model fit for RBDSQ question‐6, although AUC did not remain significant after correcting for multiple comparisons. A faster seeding profile was associated with higher question‐6 scores. RBD symptoms have been observed to fluctuate following PD diagnosis, with some individuals developing new symptoms and others experiencing a resolution over time.^30^, ^31^, ^32^ Recent work in the PPMI cohort has shown probable RBD, based on RBDSQ question‐6, is not associated with different α‐synuclein SAA parameters at baseline.^33^ Our findings may suggest that individuals with a fast seeding profile are at increased risk of developing RBD following PD diagnosis. Although further studies are required to fully explore this relationship.

This study has several limitations. Of the 396 participants with sporadic PD, 33 had missing data for their 5‐year follow‐up visit and 77 had missing data for their off‐state MDS‐UPDRS Part III. Another limitation of this study was the small number of individuals in this cohort with cognitive impairment. The median MoCA score at baseline was 28 with a median rate of change over 5 years of 0 which may result in a ceiling effect. This may reflect that those with cognitive impairment were more likely to prematurely withdraw from the study. This limits our conclusions about the association between kinetic parameters and cognitive impairment. In this study we followed participants up over 5 years however as seen in the work by Brockmann et al a longer follow‐up period (10–11 years) may be required to find differences between groups. Finally, given the small sample size for the Amprion‐24 h αS‐SAA, our analysis is likely underpowered refute associations between kinetic parameters in this assay and outcome measures.

In this study we did not find that α‐synuclein SAA parameters impact disease progression in PD. We did however find that faster kinetic profiles were associated with a greater RBDSQ question‐6 score. While the RBDSQ is validated as a screening tool rather than a severity score it assesses the most prominent features of REM sleep behavior disorder (RBD).^34^ These results indicate that SAA has an impact on features of RBD. This may suggest that SAA could play a role in prognostication for some non‐motor features of PD. Future studies are necessary to evaluate the biological and physicochemical factors influencing α‐synuclein aggregation kinetics. As α‐synuclein SAA protocols become standardized and cut‐offs for kinetic parameters are established, further exploring the prognostic utility of α‐synuclein SAA will be of great interest. As has been shown in other neurodegenerative diseases, most notably in Alzheimer's disease, it is likely that prognostication of PD will require a combination of biomarkers.^35^, ^36^ While certain biomarkers (such as α‐synuclein) may allow clinicians to diagnosis PD, others may have greater value in predicting disease severity over time.

## Author Roles

(1) Research project: A. Conception, B. Execution; (2) Statistical Analysis: A. Design, B. Execution, C. Review and Critique; (3) Manuscript: A. Writing of the first draft, B. Review and Critique.

D.B.: 1A, 1B, 2A, 2B, 3A.

C.M.: 2A, 2C, 3C.

B.H.: 2A, 2C, 3B.

J.P.B.: 2A, 2B, 2C, 3B.

A.J.N.: 1A, 1B, 2A, 2C, 3B.

C.S.: 1A, 1B, 2A, 2C, 3B.

## Disclosures

**Ethical Compliance Statement:** The PPMI study was approved by local ethics committees at each site and informed consent was obtained from participants. We confirm that we have read the Journal's position on issues involved in ethical publication and affirm that this work is consistent with those guidelines.

**Funding Sources and Conflict of Interest:** This study was supported by Barts Charity, the Michael J. Fox Foundation, Parkinson's UK, the Coordenação de Aperfeiçoamento de Pessoal de Nível Superior—Brasil (CAPES)—Finance Code 001. The authors declare that there are no conflicts of interest relevant to this work.

**Financial Disclosures for the Previous 12 Months:** B reported receiving grants from Parkinson's UK. AJN reports grants from Parkinson's UK, Barts Charity, Cure Parkinson's, National Institute for Health and Care Research, Innovate UK, the Medical College of Saint Bartholomew's Hospital Trust, Alchemab, Aligning Science Across Parkinson's Global Parkinson's Genetics Program (ASAP‐GP2) and the Michael J. Fox Foundation. He reports consultancy and personal fees from AstraZeneca, AbbVie, Profile, Bial, Charco Neurotech, Alchemab, Sosei Heptares, Umedeor and Britannia. He has share options in Umedeor. He is an Associate Editor for the *Journal of Parkinson's Disease*. CS reports grants from Innovate UK, as a co‐investigator and Michael J Fox Foundation as part of the Early‐Stage Investigators Funding Program. No other disclosures were reported.

## Supporting information


**Supplementary TABLE S1.** Reason for withdrawal from study before year 5 follow‐up visit.
**Supplementary TABLE S2.** Summary of basic and biomarker Amprion‐24 h αS‐SAA models for each primary outcome measure. Akaike information criterion (AIC), α‐synuclein SAA (α‐synuclein seeding aggregation assay), Movement Disorder Society‐Unified Parkinson's Disease Rating Scale (MDS‐UPDRS) Part III (motor section), Montreal Cognitive Assessment (MoCA), levodopa equivalent daily dose (LEDD), maximum florescence (Fmax), time to threshold (TTT), area under the curve (AUC), time to reach 50% Fmax (T50), slope (SLOPE), maximum slope (Smax), time to Smax (TSmax), neurofilament light chain (NfL).
**Supplementary TABLE S3.** Summary of basic and biomarker models for each secondary outcome measure. Akaike information criterion (AIC), α‐synuclein SAA (α‐synuclein seeding aggregation assay), Scales for Outcomes in Parkinson's disease—Autonomic Dysfunction (SCOPA‐AUT), Benton Judgment of Line Orientation (JLO), Hopkins Verbal Learning Test—Revised (HVLT‐R), Geriatric Depression Scale Short Version (GDS), REM Sleep Behavior Disorder Screening Questionnaire (RBDSQ) and Epworth Sleepiness Scale (ESS), Recognition Discrimination Index (RDI), maximum florescence (Fmax), time to threshold (TTT), area under the curve (AUC), time to reach 50% Fmax (T50), slope (SLOPE), maximum slope (Smax), time to Smax (TSmax), neurofilament light chain (NfL).

## Data Availability

The data that support the findings of this study are available from Parkinson's Progression Markers Initiative. Restrictions apply to the availability of these data, which were used under license for this study. Data are available from https://www.ppmi-info.org/ with the permission of Parkinson's Progression Markers Initiative.
