# Supplementary material for: Evaluating the Role of α‐Synuclein Seed Amplification as a Disease Progression Marker: Evidence and Uncertainties
Source: Mov Disord Clin Pract. 2025 Nov 14;13(5):1228–35. doi: 10.1002/mdc3.70434 (PMC13172763; doi:10.1002/mdc3.70434)
Supplement: Supplementary file 1 — Supplementary TABLE S1. Reason for withdrawal from study before year 5 follow‐up visit. Supplementary TABLE S2. Summary of basic and biomarker Amprion‐24 h αS‐SAA models for each primary outcome measure. Akaike information criterion (AIC), α‐synuclein SAA (α‐synuclein seeding aggregation assay), Movement Disorder Society‐Unified Parkinson's Disease Rating Scale (MDS‐UPDRS) Part III (motor section), Montreal Cognitive Assessment (MoCA), levodopa equivalent daily dose (LEDD), maximum florescence (Fmax), time to threshold (TTT), area under the curve (AUC), time to reach 50% Fmax (T50), slope (SLOPE), maximum slope (Smax), time to Smax (TSmax), neurofilament light chain (NfL). Supplementary TABLE S3. Summary of basic and biomarker models for each secondary outcome measure. Akaike information criterion (AIC), α‐synuclein SAA (α‐synuclein seeding aggregation assay), Scales for Outcomes in Parkinson's disease—Autonomic Dysfunction (SCOPA‐AUT), Benton Judgment of Line Orientation (JLO), Hopkins Verbal Learning Test—Revised (HVLT‐R), Geriatric Depression Scale Short Version (GDS), REM Sleep Behavior Disorder Screening Questionnaire (RBDSQ) and Epworth Sleepiness Scale (ESS), Recognition Discrimination Index (RDI), maximum florescence (Fmax), time to threshold (TTT), area under the curve (AUC), time to reach 50% Fmax (T50), slope (SLOPE), maximum slope (Smax), time to Smax (TSmax), neurofilament light chain (NfL). [file MDC3-13-1228-s001.docx]

Supplementary table 1:

| **Reason for withdrawal** | **n** |
| --- | --- |
| Adverse Event | 2 |
| Death | 14 |
| Lost to follow up | 11 |
| Non-compliance with study procedures | 1 |
| Institutionalized | 1 |
| Subject withdrew consent (specify) | 24 |
| Investigator decision (specify) | 1 |
| Informant / Caregiver decision (specify) | 1 |
| Other | 11 |

Reason for withdrawal from study before year 5 follow-up visit.

Supplementary table 2:

| **Outcome** | **Basic model** | | **Biomarker model** | | | | | **Likelihood ratio** | **FDR** |
| --- | --- | --- | --- | --- | --- | --- | --- | --- | --- |
|  | **Adjusted R^2^** | **AIC** | **Biomarker regression coefficient** | **Biomarker regression coefficient 95% LCI** | **Biomarker regression coefficient 95% UCI** | **Adjusted R^2^** | **AIC** |  |  |
| **Amprion-24h αS-SAA** |  | | | | | | | | |
| UPDRS-III ~ AUC | 5.08E-01 | 1.75E+02 | -4.66E-10 | -1.63E-09 | 7.00E-10 | 5.03E-01 | 1.76E+02 | 3.69E-01 | 8.62E-01 |
| UPDRS-III ~ Fmax | 5.08E-01 | 1.75E+02 | -9.78E-06 | -5.36E-05 | 3.40E-05 | 4.95E-01 | 1.77E+02 | 6.15E-01 | 8.82E-01 |
| UPDRS-III ~ Smax | 5.08E-01 | 1.75E+02 | -2.31E-02 | -1.26E-01 | 7.99E-02 | 4.96E-01 | 1.77E+02 | 6.13E-01 | 8.82E-01 |
| UPDRS-III ~ TSmax | 5.08E-01 | 1.75E+02 | 3.83E-01 | -1.96E-01 | 9.62E-01 | 5.20E-01 | 1.75E+02 | 1.41E-01 | 8.62E-01 |
| UPDRS-III ~ TTT | 5.08E-01 | 1.75E+02 | 4.04E-01 | -1.86E-01 | 9.94E-01 | 5.22E-01 | 1.74E+02 | 1.28E-01 | 8.62E-01 |
| MoCA ~ AUC | 8.73E-02 | 2.57E+02 | 1.72E-09 | -4.06E-09 | 7.51E-09 | 6.56E-02 | 2.58E+02 | 4.99E-01 | 8.82E-01 |
| MoCA ~ Fmax | 8.73E-02 | 2.57E+02 | 7.93E-05 | -1.30E-04 | 2.89E-04 | 7.37E-02 | 2.58E+02 | 3.91E-01 | 8.62E-01 |
| MoCA ~ Smax | 8.73E-02 | 2.57E+02 | 1.72E-01 | -3.36E-01 | 6.80E-01 | 6.95E-02 | 2.58E+02 | 4.42E-01 | 8.62E-01 |
| MoCA ~ TSmax | 8.73E-02 | 2.57E+02 | 1.11E-01 | -3.40E+00 | 3.62E+00 | 5.23E-02 | 2.59E+02 | 9.42E-01 | 9.91E-01 |
| MoCA ~ TTT | 8.73E-02 | 2.57E+02 | -2.55E-02 | -3.60E+00 | 3.55E+00 | 5.22E-02 | 2.59E+02 | 9.87E-01 | 9.91E-01 |
| LEDD ~ AUC | 6.28E-02 | 5.85E+02 | -9.16E-08 | -2.66E-07 | 8.28E-08 | 6.65E-02 | 5.85E+02 | 2.57E-01 | 8.62E-01 |
| LEDD ~ Fmax | 6.28E-02 | 5.85E+02 | -3.33E-03 | -9.90E-03 | 3.23E-03 | 6.45E-02 | 5.86E+02 | 2.73E-01 | 8.62E-01 |
| LEDD ~ Smax | 6.28E-02 | 5.85E+02 | -9.39E+00 | -2.51E+01 | 6.32E+00 | 7.53E-02 | 5.85E+02 | 1.98E-01 | 8.62E-01 |
| LEDD ~ TSmax | 6.28E-02 | 5.85E+02 | 7.71E+00 | -1.01E+02 | 1.17E+02 | 3.58E-02 | 5.87E+02 | 8.78E-01 | 9.91E-01 |
| LEDD ~ TTT | 6.28E-02 | 5.85E+02 | -5.63E-01 | -1.12E+02 | 1.11E+02 | 3.52E-02 | 5.87E+02 | 9.91E-01 | 9.91E-01 |

Summary of basic and biomarker Amprion-24h αS-SAA models for each primary outcome measure. Akaike information criterion (AIC), α-synuclein SAA (α-synuclein seeding aggregation assay), Movement Disorder Society-Unified Parkinson's Disease Rating Scale (MDS-UPDRS) Part III (motor section), Montreal Cognitive Assessment (MoCA), levodopa equivalent daily dose (LEDD), maximum florescence (Fmax), time to threshold (TTT), area under the curve (AUC), time to reach 50% Fmax (T50), slope (SLOPE), maximum slope (Smax), time to Smax (TSmax), neuroﬁlament light chain (NfL).

Supplementary table 3:

| **Outcome** | **Basic model** | | **Biomarker model** | | | | | **Likelihood ratio** | **FDR** |
| --- | --- | --- | --- | --- | --- | --- | --- | --- | --- |
|  | **Adjusted R^2^** | **AIC** | **Biomarker regression coefficient** | **Biomarker regression coefficient 95% LCI** | **Biomarker regression coefficient 95% UCI** | **Adjusted R^2^** | **AIC** |  |  |
| **Amprion-150h αS-SAA** |  | | | | | | | | |
| SCOPA-AUT ~ AUC | 4.15E-01 | 1.59E+03 | 9.80E-08 | -8.33E-08 | 2.79E-07 | 4.15E-01 | 1.60E+03 | 2.82E-01 | 8.62E-01 |
| SCOPA-AUT ~ Fmax | 4.15E-01 | 1.59E+03 | 5.99E-06 | -1.84E-05 | 3.04E-05 | 4.13E-01 | 1.60E+03 | 6.25E-01 | 8.82E-01 |
| SCOPA-AUT ~ SLOPE | 4.15E-01 | 1.59E+03 | -1.03E-03 | -6.11E-02 | 5.91E-02 | 4.13E-01 | 1.60E+03 | 9.73E-01 | 9.91E-01 |
| SCOPA-AUT ~ T50 | 4.14E-01 | 1.58E+03 | -2.80E-02 | -9.12E-02 | 3.52E-02 | 4.13E-01 | 1.58E+03 | 3.77E-01 | 8.62E-01 |
| SCOPA-AUT ~ TTT | 4.15E-01 | 1.59E+03 | -2.61E-02 | -8.84E-02 | 3.61E-02 | 4.14E-01 | 1.60E+03 | 4.03E-01 | 8.62E-01 |
| JLO ~ AUC | 2.81E-01 | 1.03E+03 | -2.82E-08 | -8.87E-08 | 3.23E-08 | 2.80E-01 | 1.03E+03 | 3.53E-01 | 8.62E-01 |
| JLO ~ Fmax | 2.81E-01 | 1.03E+03 | -4.63E-07 | -8.68E-06 | 7.76E-06 | 2.78E-01 | 1.03E+03 | 9.11E-01 | 9.91E-01 |
| JLO ~ SLOPE | 2.81E-01 | 1.03E+03 | -7.39E-03 | -2.75E-02 | 1.27E-02 | 2.79E-01 | 1.03E+03 | 4.63E-01 | 8.62E-01 |
| JLO ~ T50 | 2.80E-01 | 1.02E+03 | 1.31E-02 | -7.78E-03 | 3.39E-02 | 2.82E-01 | 1.02E+03 | 2.12E-01 | 8.62E-01 |
| JLO ~ TTT | 2.81E-01 | 1.03E+03 | 4.36E-03 | -1.64E-02 | 2.51E-02 | 2.78E-01 | 1.03E+03 | 6.75E-01 | 8.99E-01 |
| HVLT-R ~ AUC (Recall) | 3.63E-01 | 1.54E+03 | -3.91E-08 | -2.04E-07 | 1.26E-07 | 3.61E-01 | 1.55E+03 | 6.37E-01 | 8.82E-01 |
| HVLT-R ~ Fmax (Recall) | 3.63E-01 | 1.54E+03 | -1.24E-05 | -3.50E-05 | 1.01E-05 | 3.63E-01 | 1.54E+03 | 2.72E-01 | 8.62E-01 |
| HVLT-R ~ SLOPE (Recall) | 3.63E-01 | 1.54E+03 | -1.14E-02 | -6.65E-02 | 4.37E-02 | 3.60E-01 | 1.55E+03 | 6.81E-01 | 8.99E-01 |
| HVLT-R ~ T50 (Recall) | 3.70E-01 | 1.53E+03 | 2.04E-02 | -3.62E-02 | 7.69E-02 | 3.68E-01 | 1.53E+03 | 4.73E-01 | 8.67E-01 |
| HVLT-R ~ TTT (Recall) | 3.63E-01 | 1.54E+03 | 5.50E-03 | -5.11E-02 | 6.21E-02 | 3.60E-01 | 1.55E+03 | 8.46E-01 | 9.91E-01 |
| HVLT-R ~ AUC (Delayed Recall) | 3.59E-01 | 1.19E+03 | 5.96E-09 | -7.57E-08 | 8.76E-08 | 3.56E-01 | 1.19E+03 | 8.84E-01 | 9.91E-01 |
| HVLT-R ~ Fmax (Delayed Recall) | 3.59E-01 | 1.19E+03 | -1.34E-05 | -2.44E-05 | -2.43E-06 | 3.71E-01 | 1.19E+03 | 1.54E-02 | 3.74E-01 |
| HVLT-R ~ SLOPE (Delayed Recall) | 3.59E-01 | 1.19E+03 | -1.34E-02 | -4.05E-02 | 1.37E-02 | 3.59E-01 | 1.19E+03 | 3.25E-01 | 8.62E-01 |
| HVLT-R ~ T50 (Delayed Recall) | 3.61E-01 | 1.18E+03 | -2.76E-03 | -3.09E-02 | 2.54E-02 | 3.58E-01 | 1.18E+03 | 8.45E-01 | 9.91E-01 |
| HVLT-R ~ TTT (Delayed Recall) | 3.59E-01 | 1.19E+03 | -5.71E-03 | -3.37E-02 | 2.22E-02 | 3.57E-01 | 1.19E+03 | 6.84E-01 | 8.99E-01 |
| HVLT-R ~ AUC (RDI) | 1.07E-01 | 1.03E+03 | -3.32E-08 | -9.32E-08 | 2.68E-08 | 1.07E-01 | 1.03E+03 | 2.71E-01 | 8.62E-01 |
| HVLT-R ~ Fmax (RDI) | 1.07E-01 | 1.03E+03 | -8.57E-06 | -1.67E-05 | -4.65E-07 | 1.19E-01 | 1.03E+03 | 3.58E-02 | 5.53E-01 |
| HVLT-R ~ SLOPE (RDI) | 1.07E-01 | 1.03E+03 | -3.89E-03 | -2.39E-02 | 1.61E-02 | 1.04E-01 | 1.03E+03 | 6.98E-01 | 9.09E-01 |
| HVLT-R ~ T50 (RDI) | 1.08E-01 | 1.02E+03 | 8.22E-03 | -1.25E-02 | 2.89E-02 | 1.06E-01 | 1.03E+03 | 4.29E-01 | 8.62E-01 |
| HVLT-R ~ TTT (RDI) | 1.07E-01 | 1.03E+03 | 5.54E-03 | -1.50E-02 | 2.61E-02 | 1.04E-01 | 1.03E+03 | 5.91E-01 | 8.82E-01 |
| GDS ~ AUC | 2.37E-01 | 1.17E+03 | 4.23E-09 | -7.27E-08 | 8.12E-08 | 2.34E-01 | 1.17E+03 | 9.13E-01 | 9.91E-01 |
| GDS ~ Fmax | 2.37E-01 | 1.17E+03 | 1.16E-05 | 1.32E-06 | 2.19E-05 | 2.49E-01 | 1.17E+03 | 2.53E-02 | 5.11E-01 |
| GDS ~ SLOPE | 2.37E-01 | 1.17E+03 | 2.06E-02 | -4.89E-03 | 4.62E-02 | 2.42E-01 | 1.17E+03 | 1.08E-01 | 8.62E-01 |
| GDS ~ T50 | 2.37E-01 | 1.16E+03 | 1.74E-03 | -2.49E-02 | 2.84E-02 | 2.34E-01 | 1.16E+03 | 8.96E-01 | 9.91E-01 |
| GDS ~ TTT | 2.37E-01 | 1.17E+03 | -2.83E-03 | -2.92E-02 | 2.35E-02 | 2.34E-01 | 1.17E+03 | 8.30E-01 | 9.91E-01 |
| RBDSQ6 ~ AUC | 1.82E-01 | 7.88E+02 | 5.64E-08 | 2.07E-08 | 9.21E-08 | 2.10E-01 | 7.80E+02 | 1.81E-03 | 7.31E-02 |
| RBDSQ6 ~ Fmax | 1.82E-01 | 7.88E+02 | -2.42E-06 | -7.30E-06 | 2.46E-06 | 1.82E-01 | 7.89E+02 | 3.23E-01 | 8.62E-01 |
| RBDSQ6 ~ SLOPE | 1.82E-01 | 7.88E+02 | -1.25E-02 | -2.44E-02 | -6.33E-04 | 1.93E-01 | 7.86E+02 | 3.66E-02 | 5.53E-01 |
| RBDSQ6 ~ T50 | 1.80E-01 | 7.83E+02 | -2.20E-02 | -3.44E-02 | -9.63E-03 | 2.16E-01 | 7.73E+02 | 4.59E-04 | 2.78E-02 |
| RBDSQ6 ~ TTT | 1.82E-01 | 7.88E+02 | -2.33E-02 | -3.55E-02 | -1.12E-02 | 2.24E-01 | 7.76E+02 | 1.63E-04 | 1.97E-02 |
| ESS ~ AUC | 2.29E-01 | 1.45E+03 | -8.94E-08 | -2.23E-07 | 4.39E-08 | 2.31E-01 | 1.45E+03 | 1.82E-01 | 8.62E-01 |
| ESS ~ Fmax | 2.29E-01 | 1.45E+03 | -9.43E-06 | -2.75E-05 | 8.66E-06 | 2.29E-01 | 1.45E+03 | 2.99E-01 | 8.62E-01 |
| ESS ~ SLOPE | 2.29E-01 | 1.45E+03 | -1.74E-02 | -6.22E-02 | 2.75E-02 | 2.28E-01 | 1.45E+03 | 4.40E-01 | 8.62E-01 |
| ESS ~ T50 | 2.25E-01 | 1.44E+03 | 2.64E-02 | -1.99E-02 | 7.27E-02 | 2.26E-01 | 1.44E+03 | 2.56E-01 | 8.62E-01 |
| ESS ~ TTT | 2.29E-01 | 1.45E+03 | 2.06E-02 | -2.52E-02 | 6.64E-02 | 2.28E-01 | 1.45E+03 | 3.70E-01 | 8.62E-01 |
| **Amprion-24h αS-SAA** |  | | | | | | | | |
| SCOPA-AUT ~ AUC | 6.48E-01 | 2.51E+02 | 1.68E-10 | -2.27E-09 | 2.61E-09 | 6.38E-01 | 2.53E+02 | 8.79E-01 | 9.91E-01 |
| SCOPA-AUT ~ Fmax | 6.48E-01 | 2.51E+02 | -1.83E-06 | -9.47E-05 | 9.10E-05 | 6.38E-01 | 2.53E+02 | 9.65E-01 | 9.91E-01 |
| SCOPA-AUT ~ Smax | 6.48E-01 | 2.51E+02 | -4.30E-02 | -2.65E-01 | 1.80E-01 | 6.39E-01 | 2.53E+02 | 6.70E-01 | 8.99E-01 |
| SCOPA-AUT ~ TSmax | 6.48E-01 | 2.51E+02 | 3.72E-01 | -1.13E+00 | 1.88E+00 | 6.41E-01 | 2.52E+02 | 5.85E-01 | 8.82E-01 |
| SCOPA-AUT ~ TTT | 6.48E-01 | 2.51E+02 | 3.77E-01 | -1.16E+00 | 1.91E+00 | 6.40E-01 | 2.52E+02 | 5.89E-01 | 8.82E-01 |
| JLO ~ AUC | 5.53E-02 | 1.95E+02 | -3.80E-11 | -1.23E-09 | 1.15E-09 | 2.68E-02 | 1.97E+02 | 9.44E-01 | 9.91E-01 |
| JLO ~ Fmax | 5.53E-02 | 1.95E+02 | -2.58E-06 | -4.75E-05 | 4.23E-05 | 2.71E-02 | 1.97E+02 | 8.99E-01 | 9.91E-01 |
| JLO ~ Smax | 5.53E-02 | 1.95E+02 | -1.03E-02 | -1.18E-01 | 9.76E-02 | 2.78E-02 | 1.97E+02 | 8.32E-01 | 9.91E-01 |
| JLO ~ Tsmax | 5.53E-02 | 1.95E+02 | -7.64E-03 | -7.41E-01 | 7.26E-01 | 2.67E-02 | 1.97E+02 | 9.82E-01 | 9.91E-01 |
| JLO ~ TTT | 5.53E-02 | 1.95E+02 | -6.54E-03 | -7.57E-01 | 7.44E-01 | 2.67E-02 | 1.97E+02 | 9.85E-01 | 9.91E-01 |
| HVLT-R ~ AUC (Recall) | 3.21E-01 | 2.42E+02 | -1.30E-09 | -3.74E-09 | 1.14E-09 | 3.25E-01 | 2.43E+02 | 2.42E-01 | 8.62E-01 |
| HVLT-R ~ Fmax (Recall) | 3.21E-01 | 2.42E+02 | -5.07E-05 | -1.44E-04 | 4.29E-05 | 3.25E-01 | 2.43E+02 | 2.35E-01 | 8.62E-01 |
| HVLT-R ~ Smax (Recall) | 3.21E-01 | 2.42E+02 | -1.47E-01 | -3.68E-01 | 7.46E-02 | 3.37E-01 | 2.42E+02 | 1.48E-01 | 8.62E-01 |
| HVLT-R ~ Tsmax (Recall) | 3.21E-01 | 2.42E+02 | 1.01E-01 | -1.24E+00 | 1.44E+00 | 3.01E-01 | 2.44E+02 | 8.68E-01 | 9.91E-01 |
| HVLT-R ~ TTT (Recall) | 3.21E-01 | 2.42E+02 | 7.66E-02 | -1.30E+00 | 1.45E+00 | 3.01E-01 | 2.44E+02 | 9.02E-01 | 9.91E-01 |
| HVLT-R ~ AUC (Delayed Recall) | 3.50E-01 | 1.98E+02 | -1.23E-09 | -2.54E-09 | 7.46E-11 | 3.98E-01 | 1.96E+02 | 4.23E-02 | 5.68E-01 |
| HVLT-R ~ Fmax (Delayed Recall) | 3.50E-01 | 1.98E+02 | -2.90E-05 | -7.83E-05 | 2.04E-05 | 3.58E-01 | 1.99E+02 | 1.99E-01 | 8.62E-01 |
| HVLT-R ~ Smax (Delayed Recall) | 3.50E-01 | 1.98E+02 | -7.72E-02 | -1.93E-01 | 3.85E-02 | 3.66E-01 | 1.98E+02 | 1.45E-01 | 8.62E-01 |
| HVLT-R ~ Tsmax (Delayed Recall) | 3.50E-01 | 1.98E+02 | 2.55E-01 | -5.04E-01 | 1.01E+00 | 3.40E-01 | 2.00E+02 | 4.58E-01 | 8.62E-01 |
| HVLT-R ~ TTT (Delayed Recall) | 3.50E-01 | 1.98E+02 | 2.40E-01 | -5.37E-01 | 1.02E+00 | 3.38E-01 | 2.00E+02 | 4.96E-01 | 8.82E-01 |
| HVLT-R ~ AUC (RDI) | 1.35E-01 | 1.44E+02 | 1.01E-10 | -5.27E-10 | 7.29E-10 | 1.12E-01 | 1.46E+02 | 7.21E-01 | 9.19E-01 |
| HVLT-R ~ Fmax (RDI) | 1.35E-01 | 1.44E+02 | 7.91E-06 | -1.56E-05 | 3.14E-05 | 1.22E-01 | 1.45E+02 | 4.58E-01 | 8.62E-01 |
| HVLT-R ~ Smax (RDI) | 1.35E-01 | 1.44E+02 | 1.31E-02 | -4.30E-02 | 6.93E-02 | 1.15E-01 | 1.46E+02 | 6.05E-01 | 8.82E-01 |
| HVLT-R ~ Tsmax (RDI) | 1.35E-01 | 1.44E+02 | 1.04E-01 | -2.74E-01 | 4.83E-01 | 1.18E-01 | 1.46E+02 | 5.44E-01 | 8.82E-01 |
| HVLT-R ~ TTT (RDI) | 1.35E-01 | 1.44E+02 | 9.96E-02 | -2.88E-01 | 4.87E-01 | 1.17E-01 | 1.46E+02 | 5.71E-01 | 8.82E-01 |
| GDS ~ AUC | 2.77E-02 | 1.92E+02 | 6.36E-10 | -5.26E-10 | 1.80E-09 | 3.46E-02 | 1.92E+02 | 2.30E-01 | 8.62E-01 |
| GDS ~ Fmax | 2.77E-02 | 1.92E+02 | 9.14E-06 | -3.40E-05 | 5.23E-05 | 3.88E-03 | 1.93E+02 | 6.40E-01 | 8.82E-01 |
| GDS ~ Smax | 2.77E-02 | 1.92E+02 | 2.27E-02 | -8.07E-02 | 1.26E-01 | 4.28E-03 | 1.93E+02 | 6.28E-01 | 8.82E-01 |
| GDS ~ Tsmax | 2.77E-02 | 1.92E+02 | 1.68E-01 | -5.40E-01 | 8.75E-01 | 5.28E-03 | 1.93E+02 | 6.01E-01 | 8.82E-01 |
| GDS ~ TTT | 2.77E-02 | 1.92E+02 | 1.70E-01 | -5.54E-01 | 8.93E-01 | 5.15E-03 | 1.93E+02 | 6.04E-01 | 8.82E-01 |
| RBDSQ6 ~ AUC | 4.73E-02 | 1.36E+02 | -2.23E-11 | -5.82E-10 | 5.37E-10 | 1.87E-02 | 1.38E+02 | 9.30E-01 | 9.91E-01 |
| RBDSQ6 ~ Fmax | 4.73E-02 | 1.36E+02 | -7.66E-06 | -2.86E-05 | 1.33E-05 | 3.47E-02 | 1.38E+02 | 4.20E-01 | 8.62E-01 |
| RBDSQ6 ~ Smax | 4.73E-02 | 1.36E+02 | -1.43E-02 | -6.52E-02 | 3.67E-02 | 2.80E-02 | 1.38E+02 | 5.37E-01 | 8.82E-01 |
| RBDSQ6 ~ Tsmax | 4.73E-02 | 1.36E+02 | -2.63E-01 | -5.94E-01 | 6.89E-02 | 9.01E-02 | 1.35E+02 | 8.56E-02 | 8.62E-01 |
| RBDSQ6 ~ TTT | 4.73E-02 | 1.36E+02 | -2.83E-01 | -6.20E-01 | 5.53E-02 | 9.76E-02 | 1.35E+02 | 7.01E-02 | 7.71E-01 |
| ESS ~ AUC | -4.77E-02 | 2.33E+02 | 1.95E-09 | -1.95E-10 | 4.10E-09 | 2.41E-02 | 2.31E+02 | 4.92E-02 | 5.96E-01 |
| ESS ~ Fmax | -4.77E-02 | 2.33E+02 | 4.66E-05 | -3.93E-05 | 1.33E-04 | -4.07E-02 | 2.33E+02 | 2.33E-01 | 8.62E-01 |
| ESS ~ Smax | -4.77E-02 | 2.33E+02 | 1.03E-01 | -1.03E-01 | 3.09E-01 | -4.64E-02 | 2.34E+02 | 2.70E-01 | 8.62E-01 |
| ESS ~ Tsmax | -4.77E-02 | 2.33E+02 | -3.63E-01 | -1.78E+00 | 1.05E+00 | -7.13E-02 | 2.34E+02 | 5.70E-01 | 8.82E-01 |
| ESS ~ TTT | -4.77E-02 | 2.33E+02 | -3.03E-01 | -1.75E+00 | 1.14E+00 | -7.43E-02 | 2.35E+02 | 6.41E-01 | 8.82E-01 |
| **LogNfL** |  | | | | | | | | |
| SCOPA-AUT | 4.62E-01 | 1.65E+03 | -1.20E+00 | -3.22E+00 | 8.13E-01 | 4.63E-01 | 1.65E+03 | 2.35E-01 | 8.62E-01 |
| JLO | 2.36E-01 | 1.10E+03 | -6.50E-02 | -7.99E-01 | 6.69E-01 | 2.33E-01 | 1.10E+03 | 8.60E-01 | 9.91E-01 |
| HVLT-R (Recall) | 3.59E-01 | 1.59E+03 | -1.14E+00 | -2.97E+00 | 6.85E-01 | 3.60E-01 | 1.59E+03 | 2.13E-01 | 8.62E-01 |
| HVLT-R (Delayed Recall) | 3.56E-01 | 1.23E+03 | -1.15E+00 | -2.06E+00 | -2.50E-01 | 3.69E-01 | 1.22E+03 | 1.14E-02 | 3.46E-01 |
| HVLT-R (RDI) | 1.13E-01 | 1.02E+03 | -4.58E-01 | -1.07E+00 | 1.56E-01 | 1.17E-01 | 1.02E+03 | 1.38E-01 | 8.62E-01 |
| GDS | 1.91E-01 | 1.23E+03 | -5.07E-02 | -9.48E-01 | 8.46E-01 | 1.88E-01 | 1.23E+03 | 9.10E-01 | 9.91E-01 |
| RBDSQ6 | 1.85E-01 | 8.12E+02 | -1.67E-01 | -5.71E-01 | 2.36E-01 | 1.84E-01 | 8.13E+02 | 4.09E-01 | 8.62E-01 |
| ESS | 2.00E-01 | 1.50E+03 | -7.59E-01 | -2.29E+00 | 7.69E-01 | 2.00E-01 | 1.50E+03 | 3.22E-01 | 8.62E-01 |

Summary of basic and biomarker models for each secondary outcome measure. Akaike information criterion (AIC), α-synuclein SAA (α-synuclein seeding aggregation assay), Scales for Outcomes in Parkinson’s disease - Autonomic Dysfunction (SCOPA-AUT), Benton Judgment of Line Orientation (JLO), Hopkins Verbal Learning Test - Revised (HVLT-R), Geriatric Depression Scale Short Version (GDS), REM Sleep Behaviour Disorder Screening Questionnaire (RBDSQ) and Epworth Sleepiness Scale (ESS), Recognition Discrimination Index (RDI), maximum florescence (Fmax), time to threshold (TTT), area under the curve (AUC), time to reach 50% Fmax (T50), slope (SLOPE), maximum slope (Smax), time to Smax (TSmax), neuroﬁlament light chain (NfL).
